# Genome-wide transposon screening uncovers phage resistance mechanisms and bidirectional effects on antibiotic susceptibility in carbapenem-resistant *Klebsiella pneumoniae*

**DOI:** 10.1128/aac.00506-26

**Published:** 2026-08-05

**Authors:** Zhichen Zhu, Tengfei Long, Bingjie Wang, Weijia Ding, Yuxin Yan, Yicheng Wen, Yapin Zhao, Jie Zhu, Liang Wang, Jinnan Lv, Barry N. Kreiswirth, Hong Du, Liang Chen

**Affiliations:** 1 Department of Clinical Laboratory, The Second Affiliated Hospital of Soochow University 105860, Suzhou, Jiangsu, China; 2 Center for Discovery and Innovation, Hackensack-Meridian Health 3139, Nutley, New Jersey, USA; 3 Department of Pharmacy Practice, School of Pharmacy and Pharmaceutical Sciences, University at Buffalo 15497 , Buffalo, New York, USA; 4 Department of Clinical Laboratory, Shanghai Pulmonary Hospital, School of Medicine, Tongji University 89668, Shanghai, China; 5 Department of Clinical Laboratory, The Second People’s Hospital of Zhangjiagang 585891, Suzhou, Jiangsu, China; 6 MOE Key Laboratory of Geriatric Diseases and Immunology, The Second Affiliated Hospital of Soochow University 105860, Suzhou, Jiangsu, China; University of Pennsylvania Perelman School of Medicine, Philadelphia, Pennsylvania, USA

**Keywords:** phage therapy, transposon sequencing, phage-antibiotic synergy, carbapenem resistance, phage resistance

## Abstract

Phage therapy and phage-antibiotic combinations are promising strategies against multidrug-resistant pathogens like carbapenem-resistant *Klebsiella pneumoniae* (CRKP). However, the mechanisms underlying phage resistance and the molecular basis of the rational design of phage-antibiotic combinations require further investigation. Using genome-wide transposon screening, CRISPR-Cas9 knockout, and plasmid complementation in a clinical ST11-KL64 CRKP strain, we investigated susceptibility determinants for phage P545 and their impact on antibiotic susceptibility. We identified multiple phage resistance mechanisms, including disruption of phage receptor lipopolysaccharide (LPS) biosynthesis genes (involving deletions of *waaQ*, *wabH*, *wabG*, *ugd*, *galU*, and *wcaG*), inhibition of phage burst through an undefined pathway (involving deletion of *sirB1*), and increased mutation frequency (involving deletions of *mutS* and *mutL*). Furthermore, we found that deletions of LPS-related genes reduced resistance to multiple antibiotics, while *mutS* and *mutL* deletions increased resistance to several antibiotics. Further synergy assays showed that P545 in combination with meropenem, colistin, or ceftazidime had synergistic effects in the *in vitro* killing of host bacteria. By integrating genetic screening and functional validation, our approach offers a versatile platform for dissecting phage targets, understanding resistance mechanisms, and evaluating phage-antibiotic interactions. These findings provide valuable tools and insights for optimizing phage-phage and phage-antibiotic combination therapies against clinically significant multidrug-resistant pathogens.

## INTRODUCTION

The rapid increase of antimicrobial resistance (AMR) poses a significant threat to global public health, with carbapenem-resistant *Klebsiella pneumoniae* (CRKP) ranking among the most challenging pathogens to manage (1). CRKP infections are associated with high morbidity and mortality rates, particularly in healthcare settings, due to the limited therapeutic options available. The global dissemination of CRKP has been driven by several high-risk clones (2, 3). For instance, ST258 strains harboring capsular polysaccharides (CPS) CPS-1 (KL106) or CPS-2 (KL107) were the most common clones in the United States, while in Asia, particularly in China, ST11 strains with KL64 or KL47 capsular types have become the predominant clones (4–7).

As the efficacy of traditional antibiotics continues to decline, there is an urgent need for alternative therapeutic strategies to combat these life-threatening infections. Phage therapy, the use of bacteriophages to target and kill bacterial pathogens, has re-emerged as a promising approach to address the growing threat of antimicrobial resistance (8, 9). However, the successful and rational implementation of phage therapy relies on a deep understanding of the molecular mechanisms underlying phage-bacteria interactions. Currently, the field lacks a comprehensive understanding of how bacteria develop resistance to phages, which is critical for designing effective and mechanism-based therapeutic strategies. To address this gap, functional genomic tools such as transposon sequencing (TnSeq) can be invaluable (10) as it enables high-throughput, genome-wide screening to systematically identify the genetic determinants of phage susceptibility and resistance. These insights can inform more targeted and sustainable phage therapies, potentially enhancing their clinical efficacy.

On the other side, a phage-antibiotic combination has shown significant potential to improve treatment outcomes and reduce resistance development through evolutionary trade-off for phage resistance (11, 12). For instance, the *Pseudomonas aeruginosa* phage ΦOMKO1 uses the MexXY-OprM efflux pump as its receptor. When bacteria evolve resistance to ΦOMKO1 by inactivating or deleting this pump, they simultaneously lose a key multidrug efflux system, resulting in resensitization to several antibiotics, including ciprofloxacin (CIP), tetracycline (TC), ceftazidime (CAZ), and erythromycin (EM) (13, 14). This illustrates how the specific mechanism of phage resistance can directly reshape antibiotic susceptibility profiles, sometimes generating clinically favorable outcomes. However, other studies have reported antagonistic or even negative interactions between phages and antibiotics (15, 16), underscoring that combination outcomes are highly context-dependent. Hence, effective phage-antibiotic combinatorial strategies require rational design informed by a deeper understanding of the genetic and molecular pathways that control both phage resistance and antibiotic susceptibility, ultimately enabling more predictable and synergistic therapeutic strategies.

In our previous work, we identified a *Myovirus* phage, designated as P545 (17), which belongs to the class *Caudoviricetes* and genus *Marfavirus*. P545 exhibits broad-host antibacterial activity against clinical CRKP strains, including the ST11-KL64 and KL47 lineages. However, the bacterial phage receptors and resistance mechanisms to P545 remain unknown. In this study, we employed a functional genomic approach to investigate the genetic determinants of P545 resistance in an ST11-KL64 strain. Using genome-wide transposon screening, CRISPR-Cas9-mediated gene knockout, and plasmid complementation, we systematically identified key genes involved in phage resistance and explored their impact on antibiotic susceptibility. Our findings reveal multiple phage resistance mechanisms, including modifications to lipopolysaccharide (LPS) structures, inhibition of phage burst, and increased mutation frequency. Interestingly, these resistance mechanisms exert bidirectional effects on antibiotic susceptibility, highlighting the complex interplay between phage resistance and antibiotic efficacy.

## RESULTS

### Transposon mutagenesis of *K. pneumoniae* strain H10544

We constructed a transposon (Tn) mutant library in an ST11-KL64 CRKP strain H10544. This strain carries one chromosome (5,482,761 bp) and four plasmids (ranging from 11,970 to 164,088 bp). H10544 harbors the carbapenemase gene *bla*_KPC-2_ and exhibits resistance to most β-lactams, including carbapenems (imipenem [IMP] and meropenem [MEM]), CAZ, cefepime (FEP), ceftriaxone (CRO), and aztreonam (AZT). However, it remains susceptible to colistin (CST), tigecycline (TGC), ceftazidime-avibactam (CAZ/AVI), and cefiderocol (FDC). Genomic analysis revealed that *wcaJ*, which encodes undecaprenyl-phosphate glucose-1-phosphate transferase responsible for the first step of capsule biosynthesis, is disrupted by an insertion sequence in H10544.

To investigate genes associated with phage resistance and other fitness-related traits, it is essential to use a highly saturated Tn mutant library. In this study, we successfully generated such a library in *K. pneumoniae* strain H10544 using the Himar1 mariner transposon system. The donor strain *E. coli* WM3064, a diaminopimelic acid (DAP) auxotroph carrying the pSAM-Tac1 plasmid, was conjugated with the recipient strain H10544. The conjugation achieved a transfer efficiency of approximately 4.3 × 10^−4^ per recipient, resulting in ~5.5 × 10^5^ unique transposon insertions.

To assess the saturation of the library, TnSeq was performed on libraries grown in LB media. The experiment was conducted twice, with three and two biological replicates, respectively, resulting in five processed samples. A total of ~39.7 million reads were generated, of which ~37.8 million (95%) contained the Himar1 mariner Tn sequences and passed quality control. Approximately 87% of the filtered reads mapped to known “TA” insertion sites in the H10544 genome (Fig. S1). The insertion density across the five libraries ranged from 68% to 76%, with an average of 72%. Cumulatively, the total insertion density among the five Tn DNA libraries was ~87%. No Tn insertions were detected in the remaining 13% of TA sites across any of the five pools, which likely represent essential gene regions. These results demonstrate that the library is highly saturated.

Essentiality analysis of chromosomal genes identified 405 essential genes (based on the Gumbel and Binomial models), 4,916 non-essential genes, 230 uncertain genes, and 288 genes that were too short to analyze (Table S1). Most essential genes encode functions critical for bacterial growth and survival, including ribosomal structural components, rRNA and tRNA processing, ATP/GTP binding, cell wall organization, DNA replication, and the biosynthesis of fatty acids, LPS, peptidoglycans, and ubiquinone as well as DNA mismatch repair (MMR) system (Table S1). The number of essential genes identified was similar to previous findings from a transposon mutant library analysis of ST258 *K. pneumoniae* strain MKP103 (a derivative of KPNIH1) (18), which is phylogenetically close to the ST11 strains.

### High-throughput identification of potential phage susceptibility determinants

**Fig 1:**
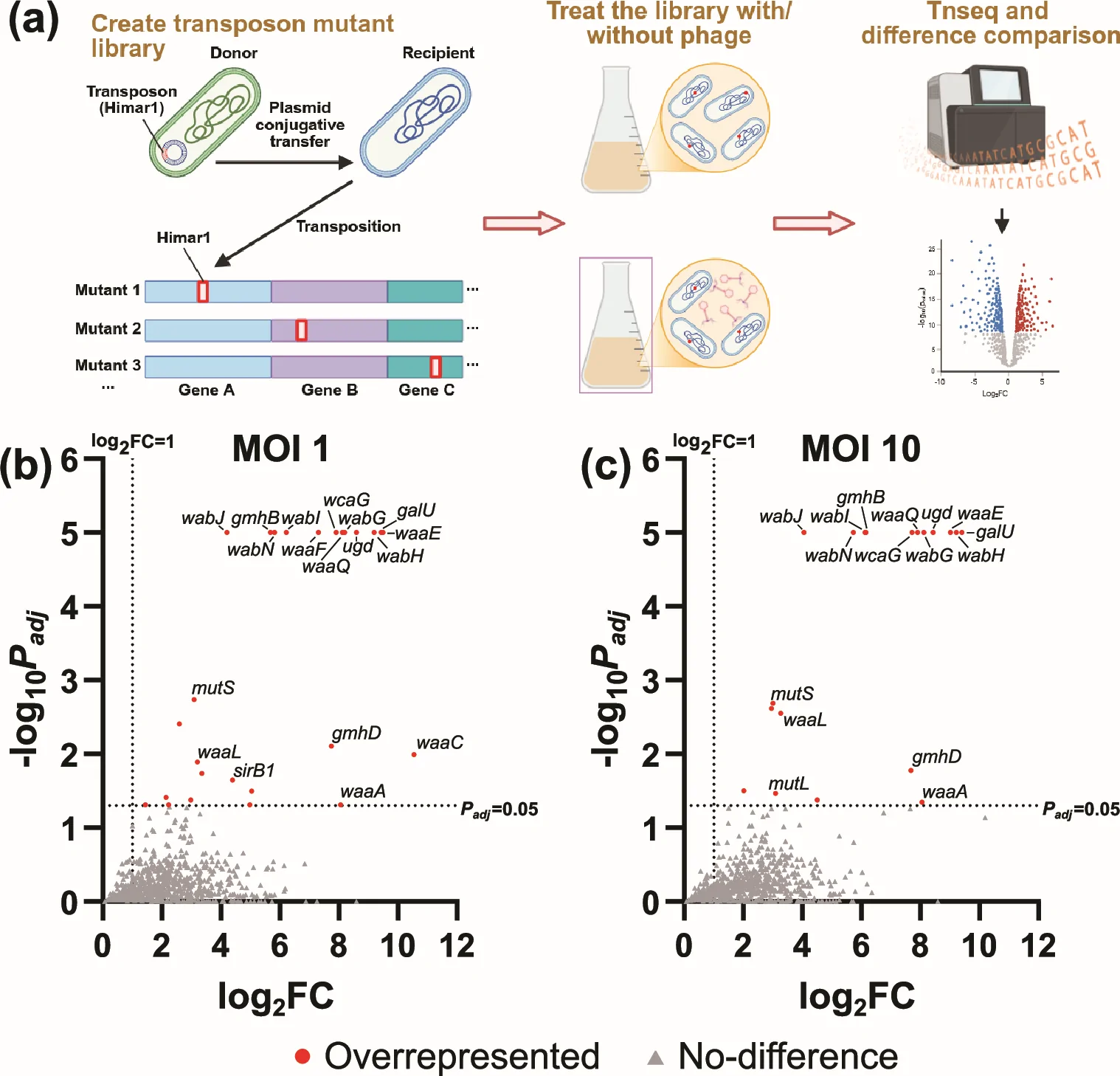
Volcano plot of overrepresented genes in H10544. (**a**) Flow chart of the genome-wide transposon screening method; the plots show the changes in the gene fitness of the transposon mutant library of H10544 following infection by (**b**) MOI 1 and (**c**) MOI 10 P545, compared to the uninfected control group (only overrepresented genes with transposon insertion are shown). To ensure visibility on the plot, *P*_adj_-values of 0 are adjusted to 0.00001. Red data points represent genes that meet the significance criteria (*Methods*) and exhibit a positive effect on phage resistance. The raw TnSeq analysis data are provided in Table S2.

Using this saturated transposon library, we assessed gene mutants conferring resistance to phage infection (Fig. 1a). The transposon mutants were challenged with phage P545 at multiplicity of infections (MOIs) of 1 and 10. The raw TnSeq analysis data have been provided in Table S2. Differentially overrepresented genes with transposon insertion are illustrated in Fig. 1b and c. The analysis identified a total of 19 candidate genes associated with increased phage resistance (Table 1).

**TABLE 1:** List of potential phage susceptibility determinants

Locus_tag^*a*^	Gene	Function	MOI 1 group		MOI 10 group		Reference
			Log_2_FC	*P*_adj_ value	Log_2_FC	*P*_adj_ value	
R5H10_00705	*waaE*	Biosynthesis of the inner core of OS	**9.45**	**0^*b*^**	**9.02**	**0**	(19)
R5H10_00710	*waaA*	Biosynthesis of the inner core of OS	**8.05**	**0.049**	**8.05**	**0.045**	(19)
R5H10_00730	*waaQ*	Biosynthesis of the inner core of OS	**8.12**	**0**	**7.91**	**0**	(19)
R5H10_00755	*waaC*	Biosynthesis of the inner core of OS	**10.54**	**0.010**	10.2	0.073	(19)
R5H10_00760	*waaF*	Biosynthesis of the inner core of OS	**7.31**	**0**	7.65	0.055	(19)
R5H10_00765	*gmhD*	Biosynthesis of the inner core of OS	**7.74**	**0.008**	**7.68**	**0.017**	(20)
R5H10_22280	*gmhB*	Biosynthesis of the inner core of OS	**5.68**	**0**	**6.15**	**0**	(21)
R5H10_00720	*wabH*	Biosynthesis of the outer core of OS	**9.19**	**0**	**9.22**	**0**	(19)
R5H10_00725	*wabG*	Biosynthesis of the outer core of OS	**8.19**	**0**	**8.12**	**0**	(19)
R5H10_00735	*wabN*	Biosynthesis of the outer core of OS	**5.82**	**0**	**5.73**	**0**	(19)
R5H10_00740	*wabJ*	Biosynthesis of the outer core of OS	**4.21**	**0**	**4.06**	**0**	(19)
R5H10_00750	*wabI*	Biosynthesis of the outer core of OS	**6.22**	**0**	**6.14**	**0**	(19)
R5H10_09005	*ugd*	Biosynthesis of the outer core of OS	**8.60**	**0**	**8.43**	**0**	(22)
R5H10_10825	*galU*	Biosynthesis of the outer core of OS	**9.43**	**0**	**9.41**	**0**	(22)
R5H10_00745	*waaL*	Biosynthesis of ligation of OS and O-antigen	**3.21**	**0.013**	**3.27**	**0.002**	(19)
R5H10_09030	*wcaG*	NAD-dependent epimerase	**7.90**	**0**	**7.72**	**0**	(23)
R5H10_10655	*sirB1*	Transcriptional regulator	**4.39**	**0.023**	3.37	0.096	(24, 25)
R5H10_05560	*mutS*	DNA mismatch repair	**3.09**	**0.002**	**3.01**	**0.002**	(26)
R5H10_24885	*mutL*	DNA mismatch repair	2.18	0.062	**3.09**	**0.034**	(26)

^*a*^ Locus_tag could be used to find the gene in H10544 (accession number CP137346).

^*b*^ *P*_adj_ values <0.05 and the corresponding Log_2_FC values are shown in bold.

Notably, 15 of these genes were involved in the biosynthesis of the core oligosaccharide (OS) of LPS in *K. pneumoniae*. These included seven genes implicated in inner OS biosynthesis (*waaA*, *waaC*, *waaE*, *waaF*, *waaQ*, *gmhB*, and *gmhD*) (19–21), seven genes associated with outer OS biosynthesis (*wabG*, *wabH*, *wabI*, *wabJ*, *wabN*, *galU*, and *ugd*) (19, 22), and one gene associated with the ligation of OS and O-antigen (*waaL*) (19). The biosynthesis of LPS is critical for maintaining the structural integrity of the outer membrane and plays a key role in bacterial resistance to phage infection by modulating receptor availability and surface accessibility. The results suggested that the LPS is most likely the P545 receptor on *K. pneumoniae*.

In addition to these definite LPS-related genes, gene *wcaG* potentially involved in LPS modification or biosynthesis was also detected. The *wcaG* gene encodes an NAD-dependent epimerase, homologous to GDP-fucose synthetase in *E. coli*, which is involved in the biosynthesis of colanic acid (CA) (23). CA repeats can participate in the formation of modified LPS, potentially altering surface properties and contributing to phage resistance. Additionally, the gene *sirB1,* associated with virulence regulation, was also identified (24, 25, 27). The *sirB1* gene encodes a putative transcriptional regulator SirB with homologs in *Salmonella typhimurium* that regulate the transcription of invasion genes (24, 25). However, the precise roles of *wcaG* and *sirB1* in *K. pneumoniae* remain less well characterized and warrant further investigation. The last two genes, *mutS* and *mutL*, are part of the MMR system in *K. pneumoniae* and are critical for maintaining genomic stability (28). MMR genes have been linked to the modulation of phage-host interactions, potentially by affecting mutation rates (26).

### Validation of potential phage susceptibility determinants

**Fig 2:**
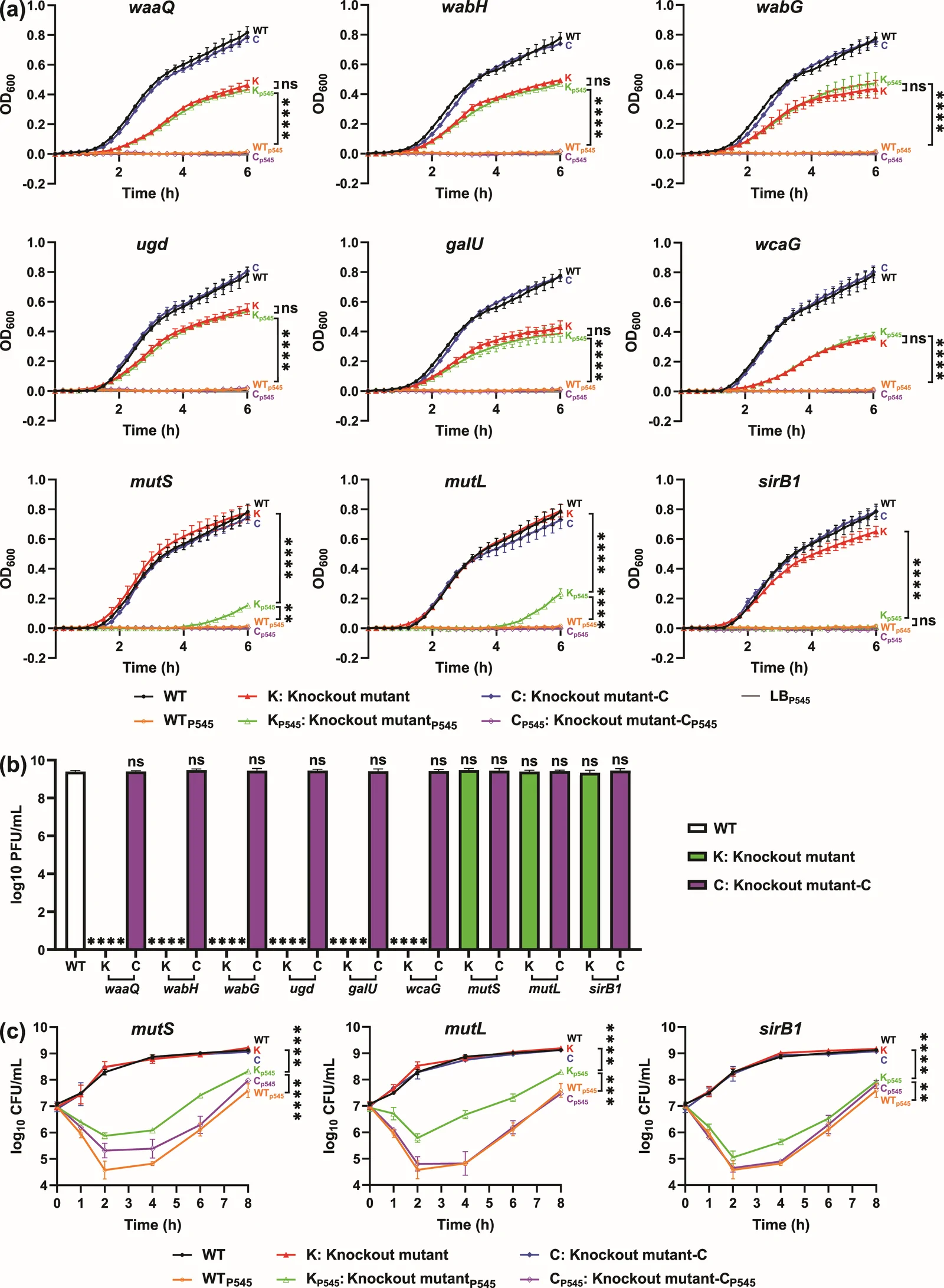
Validation of the P545 resistance phenotype of potential phage susceptibility determinants. (**a**) Growth curves of knockout mutants and complemented strains in the presence of P545. X-axis: growth time (h), Y-axis: OD_600_ value at each time point. Statistical comparisons between growth curves of different groups are annotated in the figure; (**b**) PFU when P545 was mixed with knockout mutants and complemented strains. Y-axis: log_10_-transformed values of phage titer, with statistical comparison between different groups and wild-type (WT) values labeled in the figure; (**c**) time-kill kinetics of P545 against knockout mutants and complemented strains. X-axis: killing time (h), Y-axis: log_10_-transformed bacterial count values. Statistical comparisons between time-kill curves of different groups are annotated in the figure. “Knockout mutant-C” denotes the complementation strain corresponding to the knockout mutant. “_P545_” denotes that phage P545 is added to the culture. Data are presented as mean ± SD (*n* = 3). Comparisons between curves are conducted using two-way analysis of variance (ANOVA). Statistical analysis results: No significant difference (ns) for *P*＞0.05, * *P*＜0.05，** *P*＜0.01，*** *P*＜0.001，and **** *P*＜0.0001.

To validate the function of potential phage susceptibility determinants identified through TnSeq, we selected nine genes (*waaQ*, *wabH*, *wabG*, *ugd*, *galU*, *wcaG*, *mutS*, *mutL*, and *sirB1*), which collectively cover the principal pathways implicated by the 19 candidate genes, to generate CRISPR-Cas gene knockouts followed by gene complementation using a pClone plasmid vector, in which the expression is driven by a constitutive *rpsL* promoter. Growth curve assays, plaque counting assays, and time-kill kinetics were performed to assess resistance to phage P545 (Fig. 2).

For the *waaQ*, *wabH*, *wabG*, *ugd*, *galU*, and *wcaG* gene knockout mutants, growth under P545 infection showed no significant difference compared to uninfected knockout mutants (*P* > 0.05) but was significantly enhanced relative to P545-infected wild-type (WT) (*P* < 0.0001), confirming that these knockout mutants exhibit resistant phenotypes to phage P545 (Fig. 2a). In contrast, the complemented strains under P545 infection ultimately demonstrated no statistical difference in growth curves compared to infected WT (*P* > 0.05), indicating full phenotypic reversion to WT susceptibility (Fig. 2a). Plaque counting assays further confirmed these findings that knockout mutants formed no detectable plaques, significantly fewer than those of the WT (*P* < 0.0001). In contrast, complemented strains exhibited plaque numbers similar to those of the WT (*P* > 0.05) (Fig. 2b).

For *mutS* and *mutL* genes, though the efficiency of plating (EOP) of *mutS* and *mutL* knockout mutants and complemented strains were similar to those of the WT (*P* > 0.05) (Fig. 2b), growth of knockout mutants under P545 infection remained significantly enhanced compared to the infected WT (*mutS P* < 0.01, *mutL P* < 0.0001), yet it was markedly slower than that of uninfected knockout mutants (*P* < 0.0001) (Fig. 2a). In contrast, the complemented strains under P545 infection demonstrated no statistical difference in growth curves compared to infected WT (*P* > 0.05), indicating full phenotypic reversion to WT susceptibility (Fig. 2a). Further time-kill kinetics assays demonstrated that P545 exhibited significant bactericidal activity against the Δ*mutL* knockout mutant compared to its uninfected control (*P* < 0.0001). Similarly, significant killing was also observed against the Δ*mutS* mutant (*P* < 0.0001). However, this killing effect remained attenuated relative to that of WT (*mutS P* < 0.0001; *mutL P* < 0.001) (Fig. 2c). Genetic complementation of *mutL* restored P545’s killing effect to WT levels (*P* > 0.05). In contrast, *mutS* complementation did not fully restore WT susceptibility (*P* < 0.01), though P545 killing was significantly enhanced compared to infected knockout mutants (*P* < 0.001) (Fig. 2c). These results confirm partial resistance of *mutS* and *mutL* knockout mutants to phage P545, though weaker than the complete resistance observed in the above six core OS of LPS-related gene knockout mutants.

For the *sirB1* gene, though the plaque formation efficiency of P545 in knockout mutant and complemented strains and growth curves of these strains under P545 were similar to the WT (*P* > 0.05) (Fig. 2a and b), time-kill kinetics revealed that the killing effect of P545 was weaker on *sirB1* knockout mutants compared to the WT (*P* < 0.01) (Fig. 2c). Genetic complementation of *sirB1* restored P545’s killing effect to WT levels (*P* > 0.05).

### Phage resistance via various mechanisms

**Fig 3:**
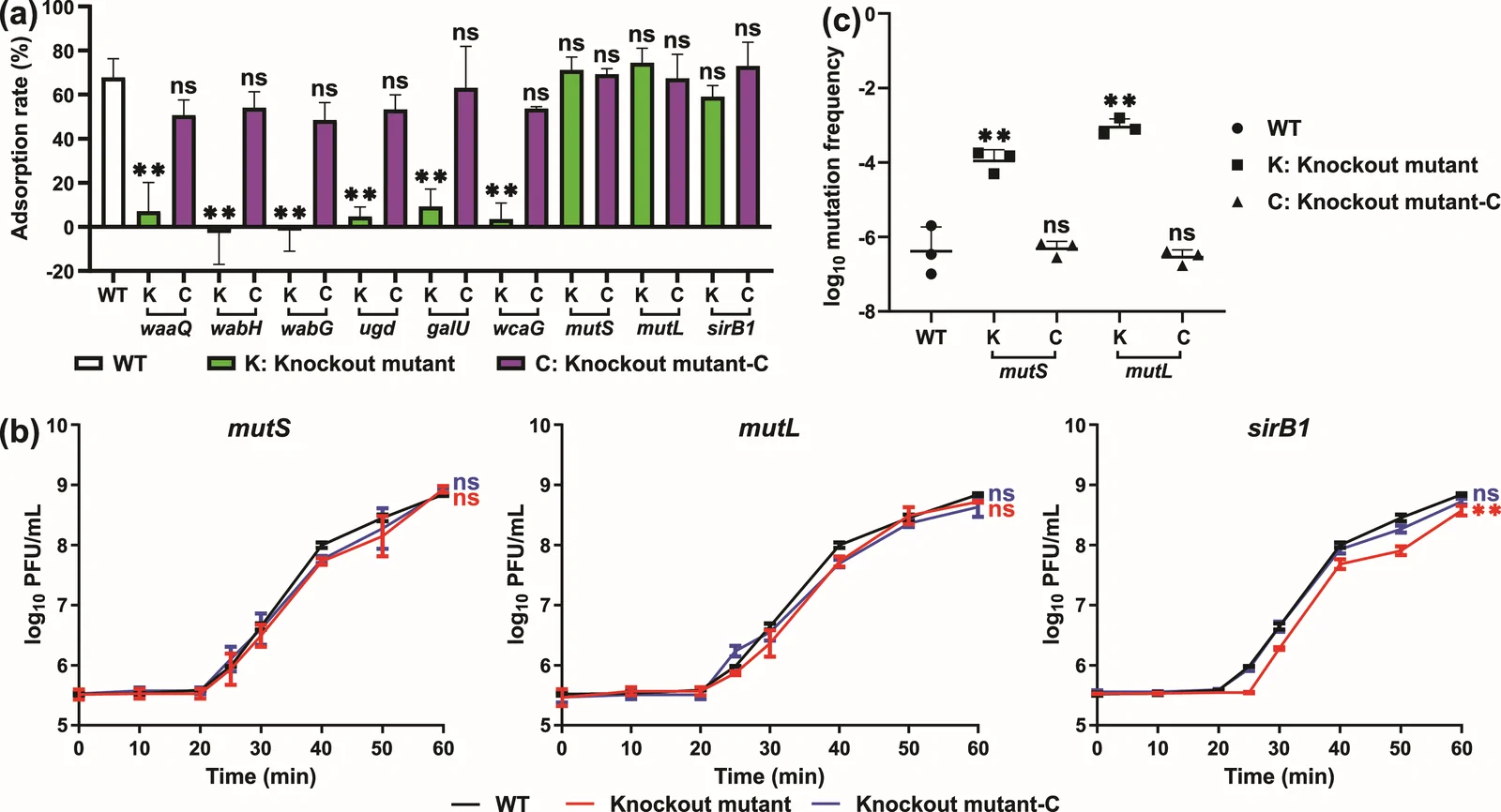
Adsorption, one-step growth, and bacterial mutation frequency toward P545. (**a**) P545 adsorption profiles with knockout mutants and complemented strains. Y-axis: phage adsorption rate. Statistical comparisons between experimental groups and WT adsorption rates are annotated in the figure; (**b**) one-step growth curves of P545 in the presence of different knockout mutants and complemented strains. X-axis: growth time (h), Y-axis: log_10_-transformed values of phage titer. Statistical comparisons between one-step growth curves of experimental groups and WT are annotated in the figure (knockout mutants in red; complemented strains in blue); (**c**) mutation frequency of knockout mutants and complemented strains to P545. Y-axis: log_10_-transformed ratio of mutant colonies on phage plates to total colonies on regular plates. Statistical comparisons between experimental groups and WT transformed values are annotated in the figure. “Knockout mutant-C” denotes the complementation strain corresponding to the knockout mutant. Data are presented as mean ± SD (*n* = 3). An unpaired *t*-test is used to analyze the statistical significance of measurement data. Comparisons between curves are conducted using ANOVA. Statistical analysis results: ns for *P*＞0.05, * *P*＜0.05，** *P*＜0.01，*** *P*＜0.001，and **** *P*＜0.0001.

We then investigated the mechanisms underlying the varying degrees of phage resistance observed within the nine selected determinants. First, to assess whether these genes are related to the defects of the phage receptor, we conducted adsorption assay (Fig. 3a). In knockout mutants for *waaQ*, *wabH*, *wabG*, *ugd*, *galU*, and *wcaG*, the phage adsorption rate was significantly lower than that of the WT strain (*P* < 0.01), confirming impaired P545 binding, while genetic complementation restored the phage adsorption rate to WT levels (*P* > 0.05). In contrast, knockout mutants for *mutS*, *mutL*, and *sirB1* showed no significant differences in phage adsorption rates compared to the WT (*P* > 0.05), suggesting that the deletion of these genes does not interfere in P545 adsorption and that resistance is mediated by other mechanisms. These findings also suggest that LPS likely serves as the receptor for P545.

Next, we examined the impact of *mutS*, *mutL*, and *sirB1* deletions on the phage proliferation of P545 through a one-step growth curve assay (Fig. 3b). The result revealed no statistical differences between *mutS*/*mutL* knockout mutants and wild-type (*P* > 0.05). In contrast, P545 proliferation in *sirB1* knockouts was significantly slower than in WT (*P* < 0.01), with an extended latent period of ~25 min compared to 20 min in WT. Genetic complementation of *sirB1* restored both latent period and proliferation kinetics to WT levels (*P* > 0.05). The data demonstrate that the deletion of the *sirB1* gene confers host resistance to P545 by inhibiting the burst of phage.

Given previous reports that *mutS* and *mutL* deletions in an ST258 *K. pneumoniae* strain led to hypermutator phenotypes (26), we explored their effects on mutation rates in the presence of phage P545 through mutation frequency assay (Fig. 3c). The result revealed that compared to WT, *mutS* and *mutL* knockout mutants exhibited significantly increased frequencies (~10^2^–10^4^) of phage-resistant mutants in the presence of phage P545 (*P* < 0.01). Additionally, genetic complementation of *mutS* and *mutL* restored mutant frequencies to WT levels (*P* > 0.05).

In summary, based on their phage resistance mechanisms post-deletion, the knockout genes in this study can be classified into three categories: (i) phage adsorption resistance (defective phage receptors), including LPS-related genes *waaQ*, *wabH*, *wabG*, *wcaG*, *ugd*, and *galU* mutants (ii), increased mutation frequency under phage pressure, including *mutS* and *mutL* mutants, and (iii) inhibition of phage burst (prolonged latent period and modest reduced proliferation rate) (Δ*sirB1*).

### Role of O-antigen in phage P545 adsorption

**Fig 4:**
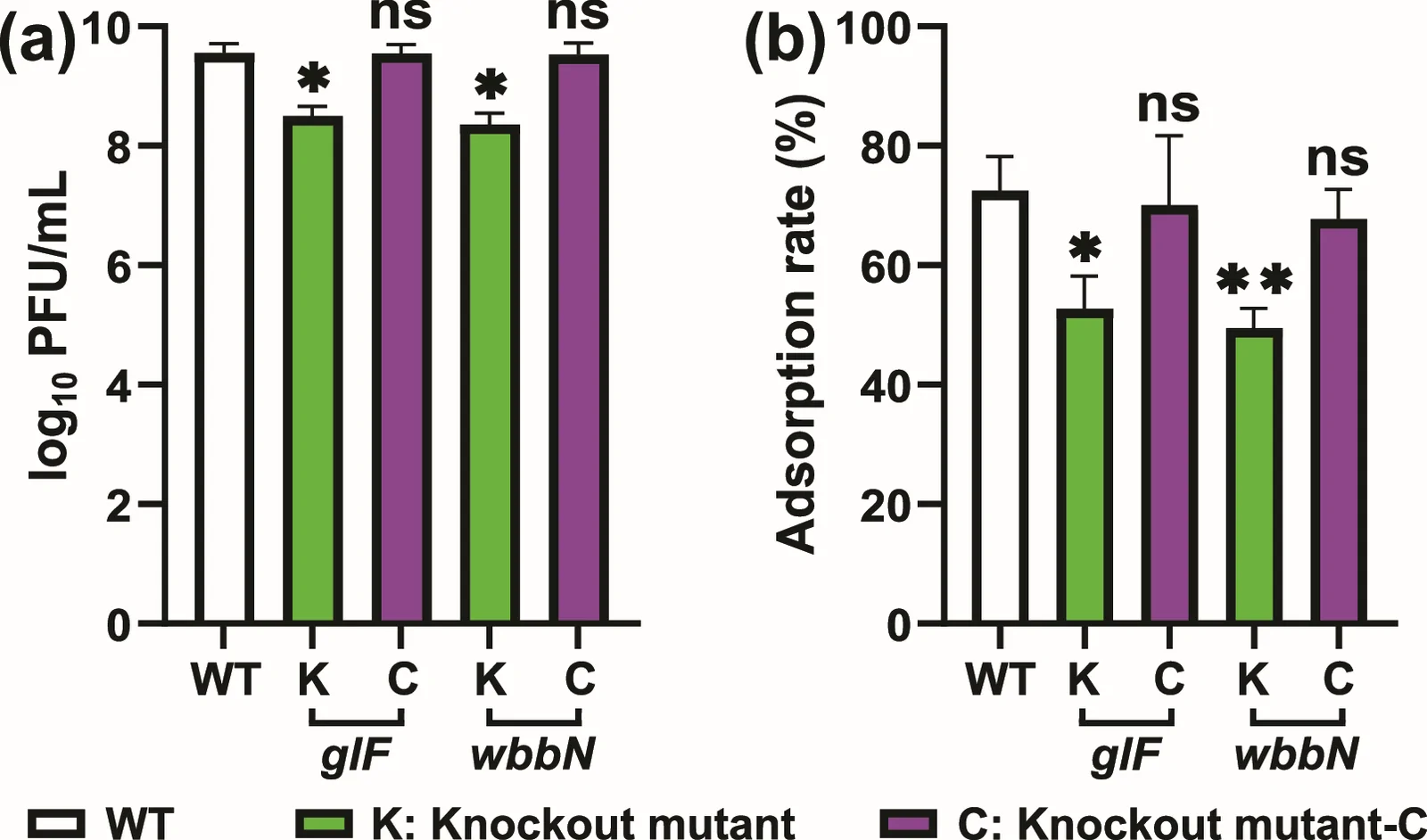
The impact of *glf* and *wbbN* deletion on P545 EOP and adsorption. (**a**) PFU when P545 was mixed with knockout mutants and complemented strains. Y-axis: log_10_-transformed values of phage titer, with statistical comparison between different groups and WT values labeled in the figure; (**b**) P545 adsorption profiles with knockout mutants and complemented strains. Y-axis: phage adsorption rate. Statistical comparisons between experimental groups and WT adsorption rates are annotated in the figure. “Knockout mutant-C” denotes the complementation strain corresponding to the knockout mutant. Data are presented as mean ± SD (*n* = 3). An unpaired *t*-test is used to analyze the statistical significance of measurement data. Statistical analysis results: ns for *P*＞0.05, * *P*＜0.05，** *P*＜0.01，*** *P*＜0.001，and **** *P*＜0.0001.

Given that defects in the core OS also compromise the assembly of outer O-antigen (29), we next determined whether the defect of O-antigen would affect phage binding. We constructed knockout mutants of *glF* and *wbbN* (genes involved in O2a-type O-antigen synthesis) (30) followed by gene complementation to define the role of O-antigen in P545 binding and resistance. The plaque counting assay result showed that the EOP of *glF* and *wbbN* knockout mutants under P545 were significantly lower to the WT strain (~ten fold; *P* < 0.05), but without conferring complete resistance to the phage. Complemented strains exhibited plaque numbers similar to WT levels (*P* > 0.05) (Fig. 4a). Further adsorption assay revealed that the phage adsorption rate of *glF* and *wbbN* knockout mutants was significantly lower than that of the WT strain (*glF P* < 0.05; *wbbN P* < 0.01), while genetic complementation restored the phage adsorption rate to WT levels (*P* > 0.05) (Fig. 4b). These findings confirmed that the O-antigen contributes, in part, to the adsorption process of P545, but its effect is weaker than that of the core OS.

### The influence of phage-resistant mutants on antimicrobial susceptibility

**Fig 5:**
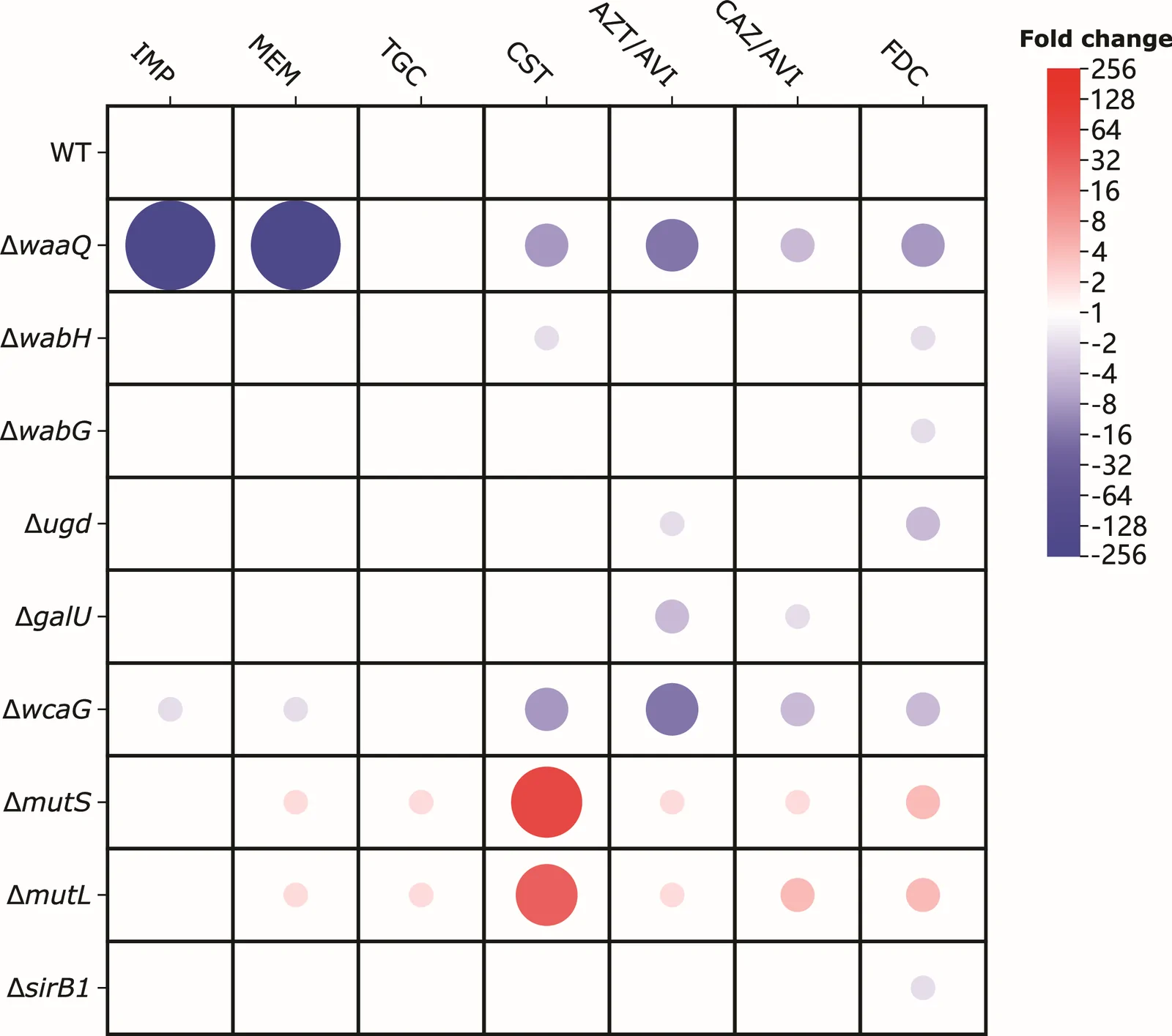
Schematic diagram of antimicrobial susceptibility of P545-resistant mutants. The fold change values represent the minimum inhibitory concentration (MIC) ratio of the knockout mutants compared to the WT strain. The raw data of antimicrobial susceptibility, including the raw MIC values for WT strain, knockout mutants, and complemented strains, are provided in Table S3. The figure is generated using Chiplot.

We next evaluated the antimicrobial susceptibility of P545-resistant mutants using the standard microdilution assay (Fig. 5; Table S3). Several last-line antimicrobials against CRKP were tested, including TGC, CST, AZT/AVI, CAZ/AVI, and FDC, as well as IMP and MEM.

Interestingly, bidirectional susceptibility changes were observed among the mutants. Compared to WT, the Δ*waaQ* and Δ*wcaG* strains exhibited most significantly reduced resistance to all tested antibiotics, except TGC. For the Δ*waaQ* strain, minimum inhibitory concentration (MIC) values of IMP and MEM decreased from 128 mg/L to ≤0.5 mg/L (≥256 fold reduction), CST from 0.5 mg/L to ≤0.0625 mg/L (≥8 fold reduction), AZT/AVI from 4/4 mg/L to ≤0.25/4 mg/L (≥16 fold reduction), CAZ/AVI from 1/4 mg/L to ≤0.25/4 mg/L (≥4 fold reduction), and FDC from 4 mg/L to ≤0.5 mg/L (≥8 fold reduction); for the Δ*wcaG* strain, MIC values of IMP and MEM decreased from 128 mg/L to 64 mg/L (2-fold reduction), CST from 0.5 mg/L to ≤0.0625 mg/L (≥8 fold reduction), AZT/AVI from 4/4 mg/L to ≤0.25/4 mg/L (≥16 fold reduction), CAZ/AVI from 1/4 mg/L to ≤0.25/4 mg/L (≥4 fold reduction), and FDC from 4 mg/L to 1 mg/L (4-fold reduction). Additionally, the *ΔwabH* strain showed reduced resistance to CST (0.5→0.25 mg/L; 2-fold reduction) and FDC (4→2 mg/L; 2-fold reduction); *ΔwabG* to FDC (4→2 mg/L; 2-fold reduction); *Δugd* to AZT/AVI (4/4→2/4 mg/L; 2-fold reduction) and FDC (4→1 mg/L; 4-fold reduction); and *ΔgalU* to AZT/AVI (4/4→1/4 mg/L; 4-fold reduction) and CAZ/AVI (1/4→0.5/4 mg/L; 2-fold reduction). In contrast, the Δ*mutS* and Δ*mutL* strains exhibited increased resistance to nearly all tested antibiotics. For the Δ*mutS* strain, the MIC value of MEM increased from 128 mg/L to 256 mg/L (2-fold increase), TGC from 1 mg/L to 2 mg/L (2-fold increase), CST from 0.5 mg/L to 32 mg/L (64-fold increase), AZT/AVI from 4/4 mg/L to 8/4 mg/L (2-fold increase), CAZ/AVI from 1/4 mg/L to 2/4 mg/L (2-fold increase), and FDC from 4 mg/L to 16 mg/L (4-fold increase); for the Δ*mutL* strain, the MIC value of MEM increased from 128 mg/L to 256 mg/L (2-fold increase), TGC from 1 mg/L to 2 mg/L (2-fold increase), CST from 0.5 mg/L to 16 mg/L (32-fold increase), AZT/AVI from 4/4 mg/L to 8/4 mg/L (2-fold increase), CAZ/AVI from 1/4 mg/L to 4/4 mg/L (4-fold increase), and FDC from 4 mg/L to 16 mg/L (4-fold increase). Genetic complementation nearly restored all MIC values to WT levels (or close to WT levels) in the aforementioned knockout strains (Table S3). To rule out the possibility that the susceptibility changes observed among the knockout strains were due to the loss of the *bla*_KPC-2_ gene during the CRISPR-Cas gene knockout process, we performed PCR and Sanger sequencing on all mutants. The results showed that *bla*_KPC-2_ remained intact in all experimental strains (data now shown). Taken together, our results highlighted the different P545 resistance mutants that could have a distinct impact on antimicrobial susceptibility.

### Phage-antibiotic synergy

**Fig 6:**
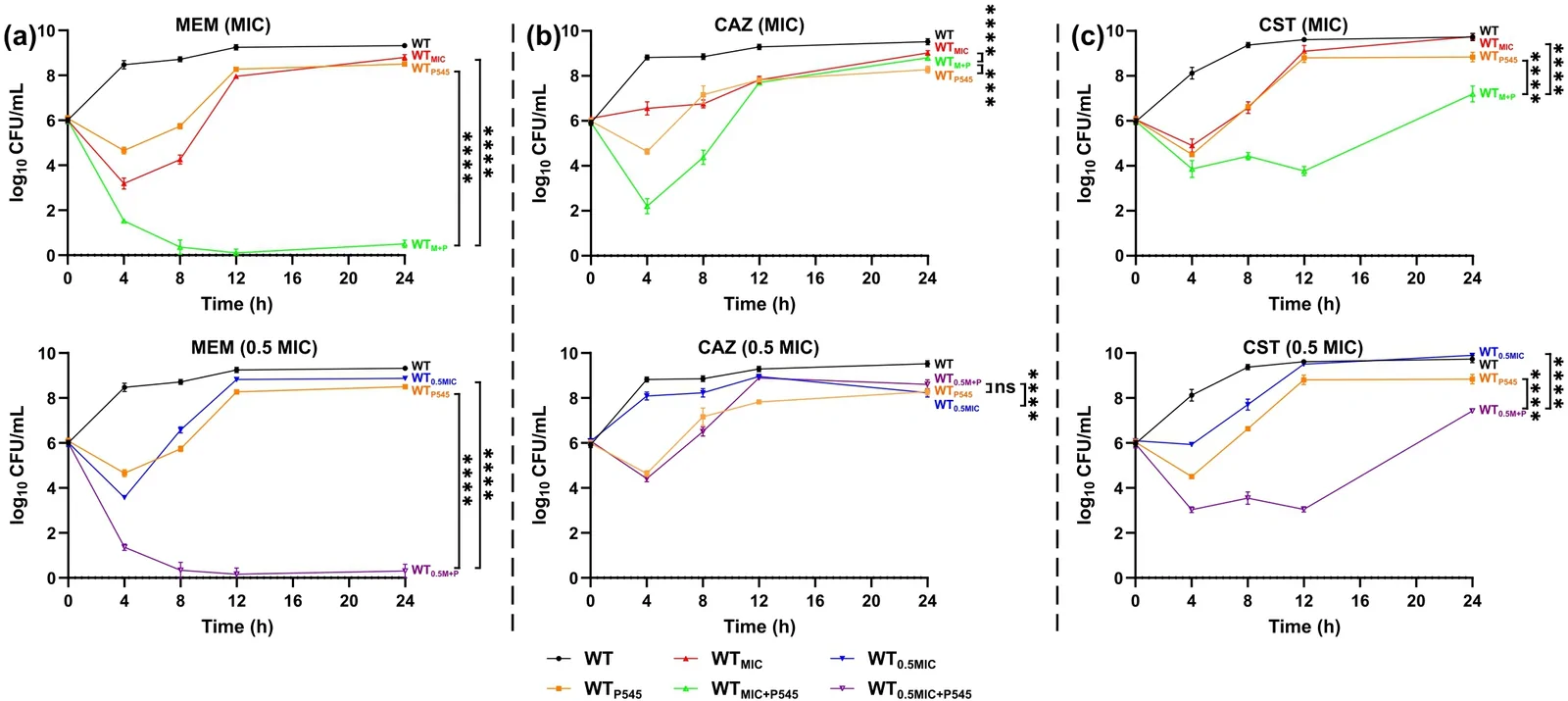
Time-kill kinetics of phage-antibiotic combination assay. Time-kill kinetics of the WT strain H10544 in the presence of P545, antibiotics: (**a**) MEM, (**b**) CAZ, (**c**) CST, and phage-antibiotic combinations. X-axis: killing time (h), Y-axis: log_10_-transformed bacterial count values. Statistical comparisons between time-kill curves of different groups are annotated in the figure. “_P545/MIC/0.5MIC_” denotes that phage P545 or/and antibiotics at appropriate concentrations is added to the culture. Data are presented as mean ± SD (*n* = 3). Comparisons between curves are conducted using ANOVA. Statistical analysis results: ns for *P*＞0.05, * *P*＜0.05，** *P*＜0.01，*** *P*＜0.001，and **** *P*＜0.0001.

The most common way for bacteria to acquire phage resistance is through the loss, mutation, or masking of phage receptors on the bacterial surface (31). Our bacterial antimicrobial susceptibility testing revealed that several phage-resistant mutants mediated by receptor loss exhibited reduced bacterial resistance to carbapenems (IMP and MEM), cephalosporins (CAZ and FDC), and CST. These findings provide a basis for the rational selection of phage-antibiotic combinations with potential synergistic effects. To investigate phage-antibiotic synergy (PAS), we conducted time-kill assays in H10544 using 1× MIC and 0.5× MIC antibiotics including MEM, CAZ, and CST and their combinations with MOI 1 P545 (Fig. 6).

For MEM and CST (Fig. 6a and c), time-kill kinetics revealed that the bactericidal efficacy of the P545 and MEM/CST (at 1× MIC or 0.5× MIC concentrations) combination was stronger than that of either the single phage or MEM/CST at corresponding concentrations (*P* < 0.0001). For CAZ (Fig. 6b), the bactericidal efficacy of the P545 and MIC CAZ (only at 1× MIC concentrations) combination was stronger than that of either the single phage or CAZ at 1× MIC concentrations (P545 *P* < 0.001; CAZ *P* < 0.0001), while the bactericidal efficacy of the P545 and CAZ (at 0.5× MIC concentrations) combination was comparable to that of P545 alone (*P* > 0.05). These results confirmed PAS between P545 and MEM, CAZ, or CST, although the efficacy differed across different combinations.

## DISCUSSION

**Fig 7:**
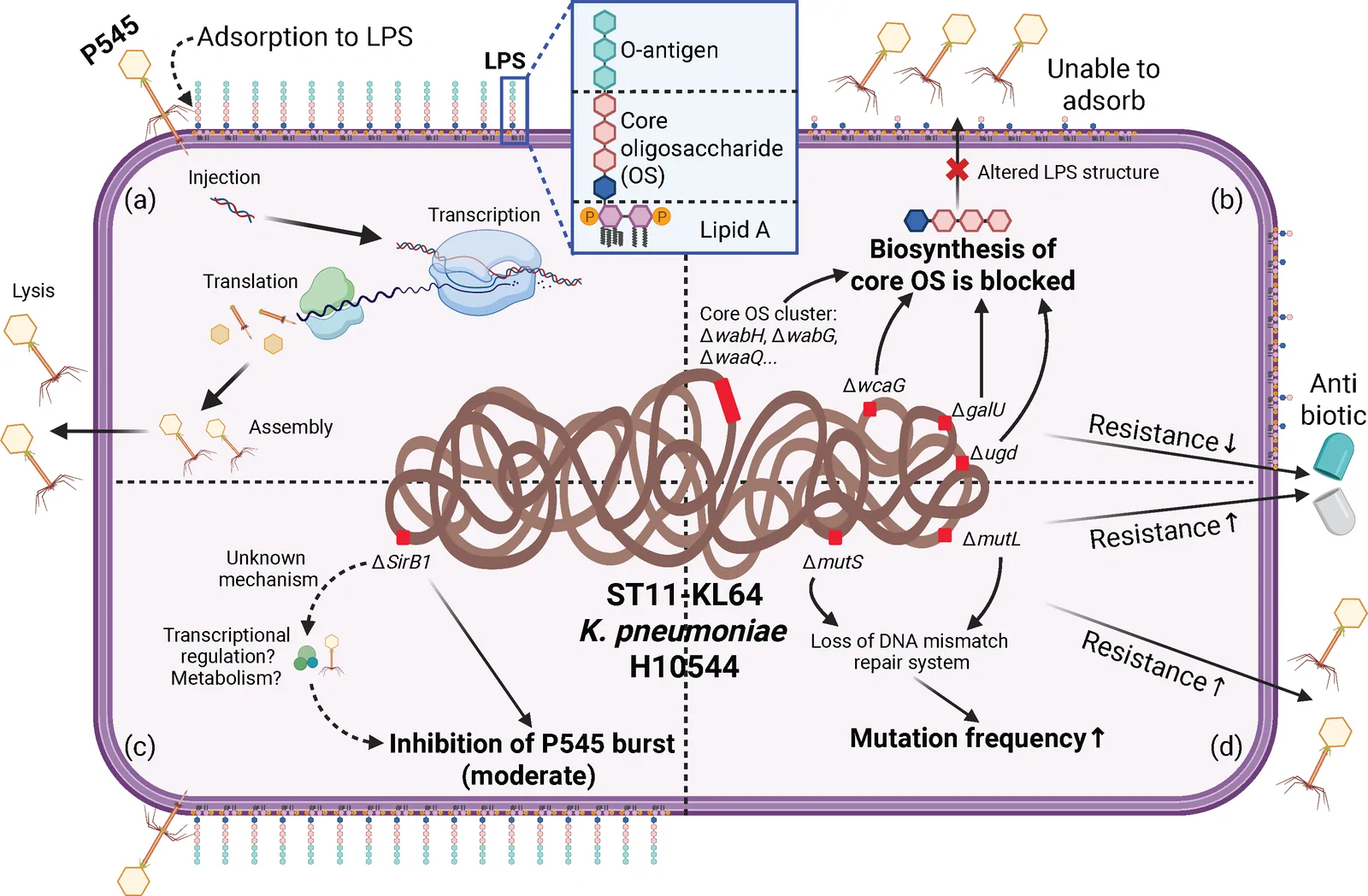
Schematic diagram of the proposed P545 resistance mechanisms in *K. pneumoniae* H10544 and their correlation with antibiotic resistance. (**a**) The life cycle of P545 in WT strains, where LPS is essential for phage adsorption; (**b**) deletion of core OS biosynthesis genes prevents phage adsorption and, in some cases, reduces antibiotic resistance; (**c**) deletion of the transcriptional regulator *sirB1* inhibits the burst of P545 through an undefined pathway; (**d**) deletion of *mutS* and *mutL*, key components of the DNA mismatch repair system, increases mutation frequency, leading to enhanced resistance against both P545 and certain antibiotics.

Phage treatment represents a promising alternative to conventional antibiotics for combating multidrug-resistant bacteria (32). However, the factors governing bacterial susceptibility to phage infection in *K. pneumoniae* require further investigation. In this study, we employed a genome-wide transposon screening approach to identify phage resistance determinants for phage P545 in the ST11-KL64 *K. pneumoniae* strain H10544 (Fig. 7). TnSeq can be used to identify both susceptibility and resistance determinants by detecting mutants that are significantly depleted or enriched following selection. In general, mutants with increased susceptibility are more readily identified under subinhibitory selection pressures, where mutants conferring increased susceptibility can be preferentially depleted while neutral and resistant mutants remain sufficiently represented in the population. However, under the relatively high-MOI conditions used in this study, a substantial number of mutants were depleted (*P*_adj_ <0.05 and log_2_ fold change ≤ −1; Table S2), likely including both bona fide susceptibility determinants and genes with neutral effects on phage susceptibility. This extensive depletion reduced our ability to reliably distinguish true susceptibility genes from the broader pool of negatively selected mutants. Consequently, our analysis was primarily focusing on identifying enriched mutants associated with phage resistance (Fig. 1).

Phage resistance mechanisms are often linked to alterations in phage receptors, such as LPS, capsules, and other bacterial surface molecules (33–35). Among the 19 candidate phage resistance determinants identified in this study, 15 genes (*waaACEFLQ*, *gmhBD*, *wabGHIJN*, *galU*, and *ugd*) were found to be involved in the biosynthesis of the core OS of LPS (19–22), while the *wcaG* gene was implicated in LPS modification or biosynthesis (23), although its precise role in *K. pneumoniae* remains uncharacterized. To further investigate their functions in phage susceptibility, six genes (*waaQ*, *wabH*, *wabG*, *galU*, *ugd*, and *wcaG*) were selected for functional validation. The results demonstrate that the deletion of these genes significantly reduces P545 adsorption to the knockout mutants, rendering complete resistance to P545 (Fig. 7a and b). Further experiments on O-antigen synthesis genes (*glf* and *wbbN*) (30) revealed that the absence of O-antigen also reduced P545 EOP and adsorption. However, these phenotypes were weaker than those caused by deletion of the core OS. This finding suggests that the O-antigen may promote binding of P545, but its effect is weaker than that of core OS.

Extensive studies have demonstrated that increased CPS production can confer resistance to LPS-targeting phages by masking phage access to the LPS receptor, as observed in *Escherichia coli* (36), *P. aeruginosa* (37), as well as *K. pneumoniae* (38). Interestingly, our reference strain H10544 contained a defective *wcaJ* gene, which encodes the enzyme catalyzing the initial step of CPS biosynthesis. Consistently, uronic acid quantification confirmed that H10544 produces extremely low levels of CPS (data not shown). To further assess the impact of CPS production on P545 activity, we performed genetic complementation of *wcaJ* in H10544 using the pClone vector, in which *wcaJ* expression was driven by a constitutive *rpsL* promoter. Following complementation, the adsorption rate of phage P545 was significantly reduced, suggesting that CPS production, when restored, could partially mask LPS and reduce phage access to its receptor (data not shown).

Beyond phage receptor-related mechanisms, deletion of *sirB1* also causes a moderate inhibition of phage P545 burst, characterized by a prolonged latent period and a slightly decreased proliferation rate (Fig. 7c). Although the underlying mechanism remains to be elucidated, these findings suggest that *sirB1* may indirectly influence phage susceptibility in *K. pneumoniae*, potentially through mechanisms distinct from those reported for its homologs in other *Enterobacteriaceae* (24, 25). Interestingly, a recent study showed that *sirB2* affects metabolic fitness under host-relevant conditions in *P. aeruginosa* (39). Whether *sirB1*-mediated modulation of P545 infection is associated with metabolic remodeling or other cellular processes remains unknown. Further mechanistic studies are needed to define the role of the *sirB* locus in phage-host interactions in *K. pneumoniae*.

In addition to LPS-related genes and the *sirB1* gene, *mutS* and *mutL*, which are part of the DNA MMR system, were identified as potential phage susceptibility determinants through TnSeq analysis. Deletion of *mutS* or *mutL* delayed bacterial killing by P545 in liquid culture, allowing the mutants to regenerate more rapidly. Mutation frequency assays revealed that Δ*mutS* and Δ*mutL* strains exhibited higher mutation rates compared to the WT strain, thereby providing a greater chance for bacteria to acquire phage resistance under selective pressure (Fig. 7d). These findings align with recent reports by Nang et al., who observed similar results in an ST258 *K. pneumoniae* exposed to phage pKMKP103_1 (26). This underscores the critical role of the MMR system, mediated by *mutS* and *mutL*, in phage resistance mechanisms in *K. pneumoniae*. Notably, clinical isolates of *E. coli*, *K. pneumoniae*, and *P. aeruginosa* with deficiencies in *mutS* and *mutL* have also been reported (40, 41), highlighting the importance of addressing hypermutation in the context of phage therapy.

PAS, a phenomenon, where the combination of phages and antibiotics is more effective at killing bacteria than either treatment alone through trade-off for phage resistance, has been widely documented (14, 42–44) and supports the use of phage-antibiotic combinations in therapy. In this study, different phage-resistant mutants exhibited diverse changes in antibiotic susceptibility. Since H10544 is a CRKP strain, we tested its susceptibility to carbapenems (IMP and MEM) and five other antibiotics commonly used against CRKP. Notably, the Δ*waaQ* and Δ*wcaG* strains showed reduced resistance to all tested antibiotics, except TGC. Similarly, deletions in *wabH*, *wabG*, *ugd*, and *galU* reduced resistance to one to two antibiotics.

Trade-offs between phage resistance and antibiotic susceptibility mediated by LPS deficiencies have been observed in multiple species. In *Yersinia pestis*, LPS core-targeting phages were selected for resistant variants with altered LPS structures that both conferred phage resistance and reduced levels of positively charged Ara4N, resulting in increased susceptibility to polymyxin B (45). In *Escherichia coli*, RfaP protein catalyzes the phosphorylation of heptose I in the LPS core, a modification that restricts the entry of hydrophobic antibiotics (46, 47). Mutations in *rfaP* led to resistance to LPS-targeting phages and increased sensitivity to hydrophobic antibiotics (48). In *K. pneumoniae*, LPS alterations that confer phage resistance have also been reported to reduce virulence (49). However, the mechanisms linking phage-mediated LPS loss to increased antibiotic sensitivity in *K. pneumoniae* remain poorly understood. One proposed explanation is that LPS deficiency enhances antibiotic penetration, as observed in other species, but this hypothesis requires further experimental validation.

It is also important to note that not all phage resistance mutations lead to a significant trade-off in antibiotic susceptibility, and some may be neutral or even increase resistance. In this study, the hypermutation phenotype of Δ*mutS* and Δ*mutL* strains facilitates the acquisition of both phage and antibiotic resistance, complicating treatment strategies involving phage-antibiotic combinations. These findings underscore the need to monitor emerging phage-resistant mutants with MMR defects in *K. pneumoniae* as these mutations can simultaneously promote resistance to both phages and antibiotics. The results also suggest that potential hypermutation inhibitors, such as nucleoside analogs (50), may act synergistically with P545 against *K. pneumoniae*, which warrants further investigation.

The genome-wide transposon screening method employed in this study proved highly effective in elucidating the interactions between phages, antibiotics, and bacteria. Among the 19 candidate phage resistance determinants identified in H10544, most were associated with phage receptors, consistent with the well-established observation that bacteria most commonly develop phage resistance through loss, mutation, or masking of phage receptors (31). As noted above, deletion of these receptor-related genes reduced H10544’s resistance to at least one of the tested antibiotics, including carbapenems (IMP and MEM), cephalosporins (CAZ and FDC), and the polypeptide antibiotic CST. Further phage-antibiotic combination assays showed PAS between P545 and MEM, CAZ, or CST, highlighting the potential of this screening method to guide clinical applications of phage-antibiotic therapies.

This study has several limitations. First, although our data strongly indicate LPS as an important determinant of P545 binding, we cannot exclude the possibility that the primary phage receptor is encoded by an essential gene that was not captured by our TnSeq screen, with LPS instead serving as a facilitating factor that promotes receptor accessibility. Direct biochemical characterization of the phage receptor is, therefore, needed to definitively establish the molecular basis of P545 attachment. Second, although we identified key bacterial genes (e.g., *sirB1*, *mutS*, and *mutL*) that influence phage susceptibility, the pathways through which they act remain incompletely defined. Similarly, while we observed strong PAS, particularly with meropenem, the molecular basis of this synergy still needs further investigation. Third, although comprehensive, the TnSeq screen captures resistance determinants under static conditions and may not reflect the dynamic co-evolution between bacteria and phages. Fourth, *in vitro* assays may not fully replicate the complex selective pressures present *in vivo*, potentially missing important fitness trade-offs. Future studies incorporating TnSeq in relevant *in vivo* models may help more accurately capture phage susceptibility determinants within the host environment. Despite these limitations, the screening and validation platform established here provides a strong foundation for dissecting phage-resistance mechanisms and advancing the development of phage-based therapeutic strategies.

In conclusion, our study reveals multiple phage resistance mechanisms in a clinical ST11-KL64 *K. pneumoniae* strain H10544 against P545 using a genome-wide transposon screening approach. Several resistance pathways exert dual effects on antibiotic susceptibility, emphasizing the importance of understanding the interplay between phages, antibiotics, and bacterial strains. Our phage screening and validation platform holds significant promise for both mechanistic studies and clinical applications. Future efforts will leverage this approach to elucidate phage-antibiotic interactions and guide the development of optimized combination therapies for multidrug-resistant infections.

## MATERIALS AND METHODS

### Bacterial strain and phage

ST11-KL64 *K. pneumoniae* H10544 was isolated from a urinary sample from a nephrology patient in Suzhou, China, in 2019. Bacterial species identification was conducted using Matrix-Assisted Laser Desorption/Ionization Time-of-Flight Mass Spectrometry (MALDI-TOF-MS). Complete genome sequencing and antimicrobial resistance gene mining of H10544 were performed as described previously (51). The complete chromosome and plasmid sequences of H10544 have been submitted to GenBank under accession numbers CP137346–CP137350.

The *Myovirus* phage P545 (169,725 bp) was also isolated in 2019 from medical sewage at a Chinese hospital (17). P545 exhibits a relatively wide host range and demonstrates antibacterial activity against 54 out of 56 clinical CRKP isolates producing KPC, NDM, and IMP, including ST11 (from three KLs of KL64, KL47, and KL25) and non-ST11 strains (ST656-KL14, ST3155-KL139, ST147-KL64, ST3369-KL116, and ST367-KL1). Detailed characteristics of P545 have been described in our previous study (17).

### Construction of the Tn mutant library

A Tn mutant library was constructed for *K. pneumoniae* H10544 using the Himar1 transposon, which randomly inserts into TA base sites. Briefly, the pSAM-Tac1 plasmid (https://figshare.com/s/18171c6d42b5ffbc1412) containing Himar1 Tn with an apramycin (APR) resistance gene was transformed from DAP auxotroph *E. coli* host WM3064 into H10544 via conjugation. Transformants were plated on LB agar containing 50 μg/mL APR and 0.1 mM IPTG (to induce the expression of Himar1 transposonase) and incubated at 37°C for 12 h to select Tn insertion mutants. Approximately 500,000 colonies were recovered and scraped from 25 150-mm Petri dishes, representing unique genome insertion sites with a coverage of 10 bp per insert. The colonies were thoroughly mixed in 10 mL of LB medium containing 30% glycerol and stored at −80°C.

### TnSeq sample preparation

Approximately 10^8^ colony-forming units (CFUs) of the *K. pneumoniae* H10544 Tn mutants were recovered from the stock and incubated in 5 mL of LB medium containing 50 μg/mL APR at 37°C overnight. The culture was then diluted to ~2.5 × 10^6^ CFU/mL in LB medium supplemented with 5 mM CaCl₂ and infected with P545 at an MOI of 1 (final titer ~2.5 × 10^6^ PFU/mL) or 10 (final titer ~2.5 × 10^7^ PFU/mL). An equal volume of phosphate-buffered saline (PBS) served as the uninfected control. Cultures were incubated at 37°C until an OD₆₀₀ = 1.0 was reached. Specific methods follow a previously published method (52).

### Sample sequencing and data analysis

Tn-adjacent DNA was generated using the NEBNext Ultra II FS DNA Library Prep Kit to construct Illumina libraries, with slight modifications from previously described methods (53). In brief, genomic DNA was extracted using the Promega SV Wizard Genomic DNA Purification Kit and quantified using both NanoDrop and Qubit analyses. Approximately 500 ng of genomic DNA was fragmented using the NEBNext Ultra II FS DNA Library Prep Kit, followed by end-repair and ligation of custom Nextera-compatible adapters containing an 8-nucleotide unique molecular identifier (UMI). A two-sided bead cleanup was performed for size selection. Transposon-genome junction enrichment was achieved through PCR using custom primers, followed by an indexing PCR using Nextera XT Index primers and a final two-sided bead cleanup. Library quality and size distribution were assessed using a Bioanalyzer High-Sensitivity DNA chip, with expected fragment sizes ranging from 250 to 550 bp. Libraries were pooled by equal mass and sequenced on Illumina platforms, with ~30% PhiX spike-in for quality control.

For data analysis, raw paired‐end sequencing reads were trimmed and quality-filtered using Trimmomatic v0.39 (54). UMI-based deduplication was conducted following methods described elsewhere (https://github.com/nppalani/TnSeq/). Reads were mapped to the genome of *K. pneumoniae* H10544 (accession number GCF_033259165.1) using the TPP program in TRANSIT v 3.3.8 (55, 56). For essential gene analysis, the TRANSIT Gumbel method was employed, while the resampling method in TRANSIT was used to identify conditionally essential genes (55). Genes with log_2_ fold change (log_2_FC) >1 and *P*_adj_ < 0.05 were identified as phage susceptibility determinants. Volcano plots were generated using GraphPad Prism 10.1.2.

### Gene knockout

Knockout mutants of *K. pneumoniae* H10544 were constructed using a two-plasmid CRISPR-Cas9 system as described previously (57), with minor modifications. Briefly, pCasKp-Apr was electroporated into H10544 and selected on LB agar plates containing 30 μg/mL apramycin (APR) at 30°C. The 20-nt base-pairing regions (N20) of the sgRNA for target genes were designed using the SSC website (58) and inserted into the *BsaI* restriction site of plasmid pSgKp-Rif (57) via Golden Gate Cloning, generating pSgKp-N20. Upstream and downstream homologous arms (400–500 bp) of the target genes were amplified by PCR and inserted into the *NotI* site of pSgKp-Rif using HiFi DNA Assembly Master Mix (NEB, USA), producing pSgKp-N20-HA. The pSgKp-N20-HA plasmids for target genes were electroporated into arabinose-induced pCasKp-Apr/H10544 competent cells. Transformants were selected on LB agar plates containing 30 μg/mL APR and 100 μg/mL rifampicin (RIF). Following PCR confirmation, single colonies were transferred to LB agar plates containing 5% sucrose and incubated at 37°C to cure the pSgKp-N20-HA and pCasKp-Apr vectors.

### Gene complementation

The coding sequences of the target genes were amplified from the *K. pneumoniae* H10544 genome using PrimeSTAR Max DNA Polymerase (Takara, Japan). The purified PCR products were subsequently cloned into the XbaI restriction site of the pClone-rpsL-Apr plasmid vector, which contains a constitutive *K. pneumoniae rpsL* promoter to control downstream gene expression, using HiFi DNA Assembly Master Mix (NEB, NY, USA). Correct constructs were identified by PCR and confirmed through Sanger sequencing. The pClone-rpsL-Apr-target vectors were then electroporated into the respective gene knockout mutants.

### Growth curve assay

The overnight bacterial culture was diluted to ~10⁸ CFU/mL (OD₆₀₀ = 0.1) and further diluted to ~10⁷ CFU/mL in LB medium supplemented with 5 mM CaCl₂ (52). Cultures were either infected with P545 at an MOI of 1 (final titer ~10^8^ PFU/mL) or treated with an equal volume of PBS (uninfected control). The total sample volume was 200 µL. Samples were transferred to a flat-bottomed 96-well microplate, with three replicates per group. Bacterial growth was monitored during incubation at 37°C using an Infinite M Plex plate reader (Tecan, Switzerland).

### Plaque counting assay

The overnight bacterial culture was diluted to OD₆₀₀ = 1 in LB medium. Ten-fold serial dilutions of phage were prepared in Saline-Magnesium Gelatin (SMG) buffer (50 mM Tris-HCl, 100 mM NaCl, 8 mM MgSO₄, and 0.01% wt/vol gelatin, at pH 7.5). A 100-μL aliquot of the diluted bacterial culture was combined with 100 μL of serially diluted phage, mixed with 5 mL of soft agar (LB medium with 0.75% wt/vol agar), and poured onto LB agar plates. Plaques were enumerated following overnight incubation at 30°C. The count was determined based on the highest dilution where a visible lysis zone was observed. Three replicates were performed for each group.

### Time-kill kinetics

The overnight bacterial culture was diluted to ~10⁸ CFU/mL (OD₆₀₀ = 0.1) and further diluted to ~10⁷ CFU/mL in LB medium. Phage P545 was added at an MOI of 10 (final titer ~10⁸ PFU/mL). The total reaction volume was 5 mL. Aliquots of 200 μL were collected at 0, 1, 2, 4, 6, and 8 h. Following ten-fold serial dilutions in PBS, 10-μL spots were plated on LB agar for viable cell counting. Three replicates were performed for each group.

### Phage adsorption assay

The overnight bacterial culture was diluted to ~10⁸ CFU/mL (OD₆₀₀ = 0.1). The culture was further diluted to ~10⁷ CFU/mL in LB medium. Phage P545 was added to reach a final titer of 2.5 × 10⁴ PFU/mL (MOI of 0.0025). At 0 and 10 min after co-incubation, 100 μL of the culture was collected and centrifuged at 16,000 × *g* for 1 min. The supernatant containing unabsorbed phage was subjected to ten-fold serial dilutions in SMG buffer. Plaque formation was performed following the same procedure as described in the plaque assay. Three replicates were conducted for each group. The phage adsorption rate was calculated using the formula: 1-[PFU of unabsorbed phage (10 min)/PFU of unabsorbed phage (0 min, total phage)].

### One-step growth curve assay

A log-phase bacterial culture was diluted to ~10⁸ CFU/mL (OD₆₀₀ = 0.1). Phage P545 was added to achieve a final titer of 10⁷ PFU/mL (MOI 0.1). After a 5-min incubation at 37°C, the culture was centrifuged at 16,000 × *g* for 1 min, and the supernatant containing free phage was removed. The bacterial pellet was washed three times with PBS and resuspended in 10 mL of LB medium. Aliquots (100 μL) were collected at 0, 10, 20, 25, 30, 40, 50, and 60 min. Each sample was serially diluted ten-fold in SMG buffer. Plaque formation was performed following the same procedure as described in the plaque assay. Three replicates were conducted for each group.

### Phage-induced mutation frequency assay

A log-phase bacterial culture was diluted to ~10⁸ CFU/mL (OD₆₀₀ = 0.1). The culture was centrifuged at 16,000 × *g* for 1 min, and the pellet was resuspended in an equal volume of PBS. The bacterial suspension was serially diluted, and 100 μL of each dilution was combined with 5 mL of soft agar (LB medium with 0.75% wt/vol agar) containing 10⁸ PFU/mL P545. The mixture was poured onto LB agar plates. Control samples without phage were included to determine the total number of colonies. Following overnight incubation at 37°C, colonies were enumerated. Mutation frequency was calculated by dividing the number of colonies formed on the agar in the experimental group over the total number of colonies.

### Phage-antibiotic combination assay

The overnight bacterial culture was diluted to ~10⁶ CFU/mL in LB medium. Treatments included MOI 1 (final titer ~10⁶ PFU/mL) of P545, 1× MIC or 0.5× MIC of the antibiotic, MOI 1 (final titer ~10⁶ PFU/mL) of P545 combined with 1× MIC or 0.5× MIC of the antibiotic, or an equal volume of PBS as a control. The total sample volume was 10 mL. Aliquots of 1 mL were collected at 0, 4, 8, 12, and 24 h for CFU counting. After centrifugation, the supernatant was discarded, and the pellet was resuspended in 100 μL PBS. Following ten-fold serial dilutions in PBS, 100 μL was plated on LB agar for viable cell counting. Three replicates were performed for each group.

### Bacterial antimicrobial susceptibility test

Antimicrobial susceptibility was assessed by determining the MICs following the 2023 Clinical and Laboratory Standards Institute (CLSI) guidelines. *E. coli* ATCC 25922 was used as the quality control strain. Three replicates were performed for each strain.

### Statistical analysis

An unpaired *t*-test was used to analyze the statistical significance of measurement data. Comparisons between curves were conducted using ANOVA. Data were analyzed using GraphPad Prism 10.1.2, with *P* < 0.05 considered statistically significant.

## Data Availability

The complete chromosome and plasmid sequences of H10544 were submitted to GenBank under accession numbers CP137346–CP137350, respectively. The TnSeq data have been deposited in BioProject PRJNA1327114. All the other data supporting the conclusions of this study are available in the paper and supplemental material.

## SUPPLEMENTAL MATERIAL

Fig. S1 aac.00506-26-s0001.pdf TnSeq genome plot of *K. pneumoniae* strain H10544.

Supplemental material aac.00506-26-s0002.docx Fig. S1 legend.

Table S1 aac.00506-26-s0003.xlsx Results of gene essentiality analysis in *K. pneumoniae* H10544.

Table S2 aac.00506-26-s0004.xlsx Raw data of TnSeq analysis.

Table S3 aac.00506-26-s0005.xlsx Antimicrobial susceptibility of P545-resistant mutants.

ASM does not own the copyrights to Supplemental Material that may be linked to, or accessed through, an article. The authors have granted ASM a non-exclusive, world-wide license to publish the Supplemental Material files. Please contact the corresponding author directly for reuse.
