# Supplementary material for: Genome-wide transposon screening uncovers phage resistance mechanisms and bidirectional effects on antibiotic susceptibility in carbapenem-resistant Klebsiella pneumoniae
Source: Antimicrob Agents Chemother. 2026 Aug 5;70(9):e00506-26. doi: 10.1128/aac.00506-26 (PMC13713007; doi:10.1128/aac.00506-26)
Supplement: Supplementary file 1 [file aac.00506-26-s0001.pdf]

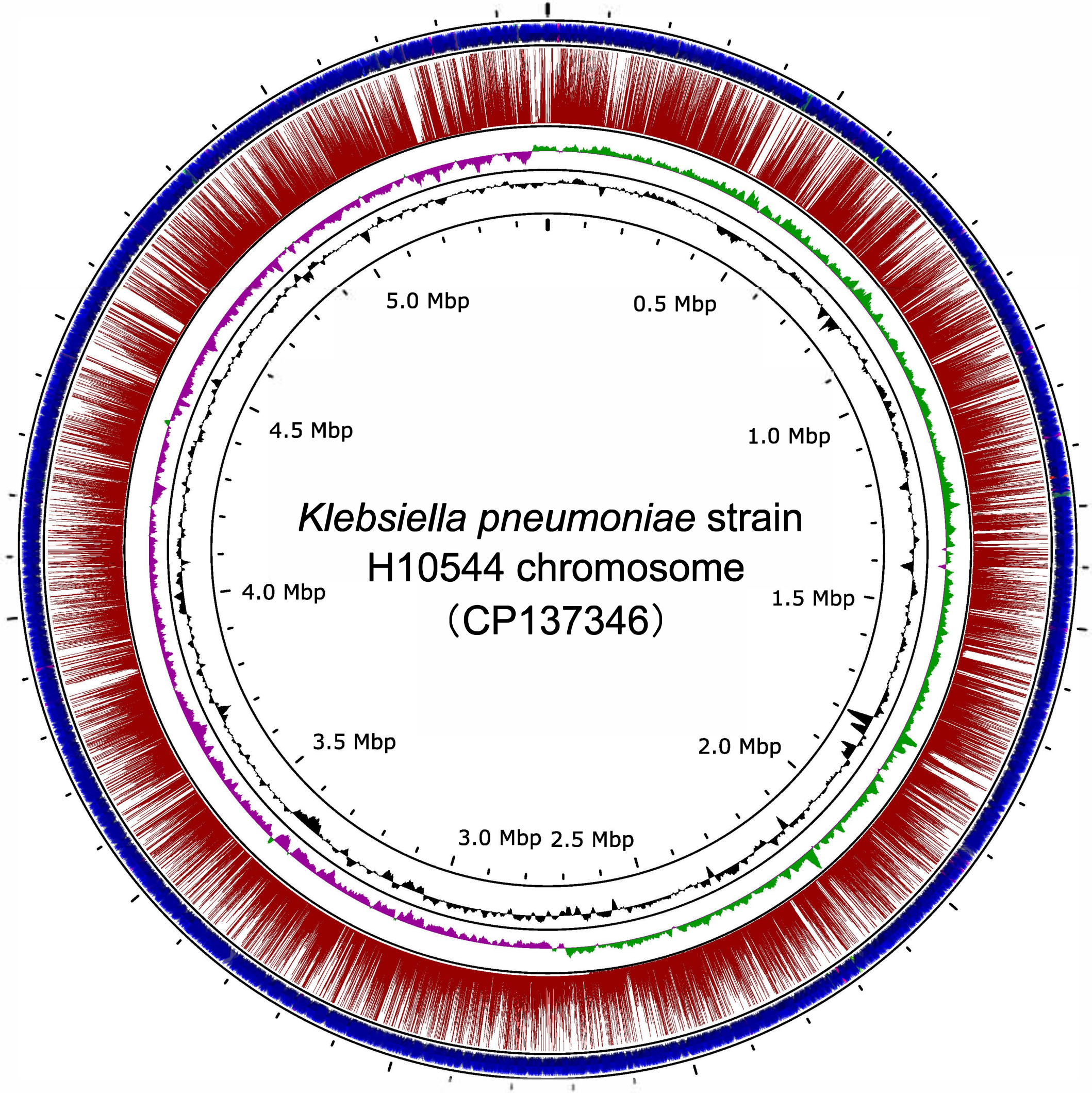
Klebsiella pneumoniae strain
H10544 chromosome
（CP137346）
0.5 Mbp
1.0 Mbp
1.5 Mbp
2.0 Mbp
2.5 Mbp
3.0 Mbp
3.5 Mbp
4.0 Mbp
4.5 Mbp
5.0 Mbp
