# Supplementary material for: Genome-wide transposon screening uncovers phage resistance mechanisms and bidirectional effects on antibiotic susceptibility in carbapenem-resistant Klebsiella pneumoniae
Source: Antimicrob Agents Chemother. 2026 Aug 5;70(9):e00506-26. doi: 10.1128/aac.00506-26 (PMC13713007; doi:10.1128/aac.00506-26)
Supplement: Supplementary file 2 [file aac.00506-26-s0002.docx]

**Fig. S1 TnSeq genome plot of *K. pneumoniae* strain H10544.** From the center outward, the circles represent the genome scale, GC content, GC skew, reads mapped to TA sites, and coding sequences (CDSs). The figure was generated using Proksee.
